# Supplementary material for: Assessing the impact of non-pharmaceutical interventions (NPI) on the dynamics of COVID-19: A mathematical modelling study of the case of Ethiopia
Source: PLoS One. 2021 Nov 16;16(11):e0259874. doi: 10.1371/journal.pone.0259874 (PMC8594814; doi:10.1371/journal.pone.0259874)
Supplement: S1 File — (PDF) [file pone.0259874.s001.pdf]

Supplementary Material

Assessing the impact of non-pharmaceutical interventions (NPI) on the dynamics of COVID-19: A mathematical modelling study in the case of Ethiopia

Bedilu Alamirie Ejigu[1*], Manalebish Debalike Asfaw [2], Lisa Cavalerie [3,4], Tilahun Abebaw[2], Mark Nanyingi [3,5], Matthew Baylis[3]

[1] Department of Statistics, College of Natural and Computational Sciences, Addis Ababa University, Addis Ababa, Ethiopia.
[2] Department of Mathematics, College of Natural and Computational Sciences, Addis Ababa University, Addis Ababa, Ethiopia.
[3] Department of Livestock and One Health, Institute of Infection, Veterinary and Ecological Sciences, University of Liverpool, Liverpool, United Kingdom
[4] International Livestock Research Institute, Addis Ababa, Ethiopia
[5] Department of Epidemiology and Public Health, School of Public Health, University of Nairobi, Kenya

* Corresponding author
E-mail: bedilu.alamirie@aau.edu.et (B.A Ejigu)

Well-posedness of the System and sensitivity analysis

To check the mathematical and biological meaningfulness of the model, both positivity and boundedness properties of the proposed model are assessed as follows. Let the domain space be defined by

$$\Omega = \left\{(S, E, I_s, I_a, H_c, IH_m, R, D, E_{nv}) \in \mathbb{R}^9_+\right\}$$

Theorem 2.1
$int(\Omega) = \{(S, E, I_s, I_a, H_c, IH_m, R, D, E_{nv})|S > 0, E \geq 0, I_s \geq 0, I_a \geq 0,$
$H_c \geq 0, IH_m \geq 0, R \geq 0, D \geq 0, E_{nv} \geq 0)\}$ is the positive invariant set of the system (Eqn 1).

Proof.

To show Ω positive, first we show S of the model is positive for all $t \geq 0$. To prove by contradiction: suppose, $S(t) = 0, S'(t) < 0, E \geq 0, I_s \geq 0, I_a \geq 0,$
$H_c \geq 0, IH_m \geq 0, R \geq 0, D \geq 0, E_{nv} \geq 0 t > 0$. Then, using the first equation of system (Eqn 1) we have,

$$\frac{dS}{dt}(t) = \pi > 0$$

which contradicts $S'(t) < 0$. Thus, S(t) remains positive for $t \geq 0$. For the rest of population variables we can show positivity using Gronwall's inequality as follows.

$$\frac{dE}{dt} \quad = \quad \gamma S - \sigma E - \mu E > -(\sigma + \mu)E. \tag{1}$$

Since $S(t) > 0$ for $t \geq 0$ we can use Gronwalls inequality and $E \geq E_0 e^{-(\sigma+\mu)t} > 0$.

$$\frac{dI_a}{dt} = (1-\theta)\sigma E - \rho I_a - \varepsilon I_a - yI_a - \mu I_a > -(\rho+\varepsilon+y+\mu)I_a \tag{2}$$

since $E(t) > 0$ for $t \geq 0$ we can use Gronwalls inequality and $I_a \geq I_{a0} e^{-(\rho+\varepsilon+y+\mu)t} \geq 0$

$$\frac{dI_s}{dt} = \theta\sigma E + \varepsilon I_a - eI_s - \omega I_s - \alpha_1 I_s - \mu I_s > -(e+\omega+\alpha_1+\mu)I_s \tag{3}$$

since $E(t) > 0, I_a(t) > 0$ for $t \geq 0$ we can use Gronwalls inequality and $I_s \geq I_{s0} e^{-(e+\omega+\alpha_1+\mu)t} \geq 0$.

$$\frac{dIH_m}{dt} = \rho I_a + eI_s - xIH_m - iIH_m - \mu IH_m > -(x+i+\mu)IH_m. \tag{4}$$

Since $I_a(t) > 0, I_s(t) > 0$ for $t \geq 0$ we can use Gronwalls inequality and $IH_m \geq IH_{m0} e^{-(x+i+\mu)t} \geq 0$.

$$\frac{dH_c}{dt} = xIH_m - gH_c - \alpha_2 H_c - \mu H_c > -(g+\alpha_2+\mu)H_c \tag{5}$$

since $I_s(t) > 0, IH_m(t) > 0$ for $t \geq 0$ we can use Gronwalls inequality and $H_c \geq H_{c0} e^{-(g+\alpha_2+\mu)t} \geq 0$

$$\frac{dR}{dt} = gH_c + iIH_m + yI_a + \omega I_s - \mu R \geq -\mu R \tag{6}$$

since $I_a(t) > 0, I_s(t) > 0, IH_m(t) > 0, H_c(t) > 0$ for $t \geq 0$ and $R(t) \geq R_0 e^{-\mu t} \geq 0$

$$\frac{dD}{dt} = \alpha_1 I_s + \alpha_2 H_c > 0 \tag{7}$$

since $I_s(t) > 0$ $H_c(t) > 0$ for $t \geq 0$ and $D \geq D_0 \geq 0$
$\dot{E}_{nv} = aI_a + bI_s - \varphi E_{nv} \leq (a+b)\overline{N} - \varphi E_{nv}$. Since $I_a(t)$ and $I_s(t)$ are less than the total population N given as $\frac{\pi}{\mu}$ for all $t \geq 0$. Applying again the Gronwall inequality, for $0 \leq E_{nv}(0) \leq \frac{a+b}{\varphi}\frac{\pi}{\mu}$ leading to

$$0 \leq E_{nv}(t) \leq \frac{a+b}{\varphi}\frac{\pi}{\mu}$$

Hence all are non-negative for $t \geq 0$. Finally, the total number of population

$$N'(t) = S'(t) + E'(t) + I_a'(t) + I_s'(t) + IH_m'(t) + H_c'(t) + R'(t) + D'(t) \tag{8}$$
$$= \pi - \mu N \tag{9}$$
$$N(t) = \frac{\pi - Ze^{-\mu t}}{\mu}. \tag{10}$$

Thus for initial data $0 < N(0) < \frac{\pi}{\mu}$ we have $0 \leq N(t) \leq \frac{\pi}{\mu}$. Moreover, for the environment E_{nv}, we have $E_{nv}' = aI_a + bI_s - \varphi E_{nv} \leq (a+b)N - \varphi E_{nv}$ Since $I_a(t)$ and $I_s(t)$ are less than N for all $t \geq 0$ applying Gronwalls inequality for $0 \leq E_{nv}(0) \leq \frac{(a+b)}{\varphi}\frac{\pi}{\mu}$ gives $0 \leq E_{nv}(t) \leq \frac{(a+b)}{\varphi}\frac{\pi}{\mu}$.

Sensitivity analysis of model parameters

In order to identify parameters which significantly influence the model system, sensitivity analysis was performed. The uncertainty and sensitivity analysis is done by using Partial Rank Correlation Coefficient (PRCC) analysis with N=10,000 samples for various input parameters [1, 4]. The partial derivative of the threshold value R_o with respect to the input parameters were computed by varying the parameters (given in Table 1) within a certain interval.

System Equilibria and Stability of the System

Disease Free Equilibrium

Equilibrium points are those points where each equation in the system in Eqn 1 is equal to zero. Equating the right hand side of each equation (Eqn 1) to zero will provide disease-free and endemic equilibrium points. In this section, the disease-free equilibrium point is described as follows.

When there is no COVID-19 in the population, the disease-free equilibrium point is given by

$$\mathcal{E}_0 = (S, E, I_a, I_s, IH_m, H_c, R, D, E_{nv}) = (\frac{\pi}{\mu}, 0, 0, 0, 0, 0, 0, 0, 0) \tag{11}$$

In this study, to compute the basic reproduction number, [3], next-generation method for the disease-free equilibrium is employed. The basic reproduction number is the average number of secondary infection cases produced in a completely susceptible population by a typical infectious individual. According to the definitions stated in [3] and [2], in the next-generation method, R_0 is the spectral radius of the next-generation matrix which is given by

$$R_0 = \rho(FV^{-1})$$

where F is the Jacobian of the rate of appearance of new infections in the infected compartment, denoted by f and V is the Jacobian of $v = v^- - v^+$ that represents all infection transfer interactions into (v^+) and out (v^-) of these compartments.

The infection compartments of our model are $(E, I_a, I_s, IH_m, H_c, E_{nv})$

$$f = \begin{pmatrix} \gamma S \\ 0 \\ 0 \\ 0 \\ 0 \\ 0 \end{pmatrix}, \qquad v = \begin{pmatrix} (\sigma + \mu)E \\ -(1-\theta)\sigma E + (\varepsilon + y + \mu)I_a \\ -\theta\sigma E - \varepsilon I_a + (e + \alpha_1 + \omega + \mu)I_s \\ -\rho I_a - eI_s + (i + x + \mu)IH_m \\ -xIH_m + (g + \alpha_2 + \mu)H_c \\ -aI_a - bI_s + \varphi E_{nv} \end{pmatrix} \tag{12}$$

Computing the Jacobian of f and evaluating it at the disease-free equilibrium point using the force of infection, we have,

$$F(\mathcal{E}_0) = \begin{pmatrix} 0 & k_4\beta_1\eta_a\frac{\mu}{\pi} & k_4\beta_1\frac{\mu}{\pi} & 0 & 0 & k_4\beta_2\frac{\varphi}{K(a+b)} \\ 0 & 0 & 0 & 0 & 0 & 0 \\ 0 & 0 & 0 & 0 & 0 & 0 \\ 0 & 0 & 0 & 0 & 0 & 0 \\ 0 & 0 & 0 & 0 & 0 & 0 \\ 0 & 0 & 0 & 0 & 0 & 0 \end{pmatrix} \tag{13}$$

and the Jacobian of v evaluated at the disease free equilibrium point using the force of infection is given by the matrix

$$V(\mathcal{E}_0) = \begin{pmatrix} \sigma+\mu & 0 & 0 & 0 & 0 & 0 \\ -(1-\theta)\sigma & (\varepsilon+y+\mu) & 0 & 0 & 0 & 0 \\ -\theta\sigma & -\varepsilon & e+\alpha_1+\omega+\mu & 0 & 0 & 0 \\ 0 & -\rho & -e & i+x+\mu & 0 & 0 \\ 0 & 0 & 0 & -x & g+\alpha_2+\mu & 0 \\ 0 & -a & -b & 0 & 0 & \varphi \end{pmatrix}. \quad (14)$$

Finding the inverse of V and computing the product FV^{-1}, the next generation matrix is given by

$$FV^{-1} = \begin{pmatrix} A & B & C & 0 & 0 & D \\ 0 & 0 & 0 & 0 & 0 & 0 \\ 0 & 0 & 0 & 0 & 0 & 0 \\ 0 & 0 & 0 & 0 & 0 & 0 \\ 0 & 0 & 0 & 0 & 0 & 0 \\ 0 & 0 & 0 & 0 & 0 & 0 \end{pmatrix} \quad (15)$$

where for

$$k_1 = \sigma+\mu, k_2 = \rho+\varepsilon+y+\mu \text{ and }, k_3 = e+\alpha_1+\omega+\mu. \quad (16)$$

$$\begin{aligned} A &= r_4\beta_1\frac{\mu}{\pi}\left(\frac{(1-\theta)\varepsilon\sigma+\theta\sigma k_2}{k_1k_2k_3}\right)+\frac{r_4\beta_1\eta_a\mu(1-\theta)\sigma}{\pi k_1k_2}+\frac{r_4\beta_2}{K}\left(\frac{b\theta\sigma k_2+(1-\theta)\sigma(\varepsilon b+ak_3)}{K\varphi k_1k_2k_3}\right) \\ B &= \frac{r_4\beta_1\varepsilon\mu}{\pi k_2k_3}+\frac{r_4\beta_1\eta_a\mu}{\pi k_2}+\frac{r_4\beta_2}{K}\left(\frac{\varepsilon b+ak_3}{\varphi k_2k_3}\right) \\ C &= \frac{r_4\beta_1\mu}{\pi k_3}+r_4\beta_2\frac{b}{K\varphi k_3} \\ D &= \frac{r_4\beta_2}{K\varphi} \end{aligned}$$

The corresponding Spectral radius of the matrix FV^{-1} is given by $R_0 = \rho(FV^{-1}) = A$, where

$$A = \underbrace{r_4\beta_1\frac{\mu}{\pi}\left(\frac{(1-\theta)\varepsilon\sigma+\theta\sigma k_2}{k_1k_2k_3}\right)}_{R_{0s}}+\underbrace{\frac{r_4\beta_1\eta_a\mu(1-\theta)\sigma}{\pi k_1k_2}}_{R_{0a}}+\underbrace{\frac{r_4\beta_2}{K}\left(\frac{b\theta\sigma k_2+\epsilon\sigma(\varepsilon b+ak_3)}{K\varphi k_1k_2k_3}\right)}_{R_{0Env}} \quad (17)$$

which can be expressed as $A = R_0 = R_{0a} + R_{0s} + R_{0Env}$ and the quantity R_0 is the basic reproduction number of the Model (Eqn 1). The quantity R_0 is the sum of the constituent reproduction numbers associated with the number of new COVID-19 cases generated by symptomatically infectious humans (R_{0s}), asymptomatically infectious humans (R_{0a}) and contaminated environment ($R_{0E_{nv}}$).

Theorem 2.2 The disease-free equilibrium (DFE) of model (Eqn 1, in the main manuscript) is locally asymptotically stable if $R_0 < 1$, and unstable if $R_0 > 1$.

Proof. It follows from Theorem 2 of [2]

The implication of Theorem 2.2 is that a small introduction of COVID-19 cases will not generate a COVID-19 outbreak if the basic reproduction number (R_0) is less than unity.

Theorem 2.3 The disease-free equilibrium of model (Eqn 1, in the main manuscript) is globally asymptotically stable if $R_0 \leq 1$.

Endemic Equilibrium

The endemic equilibrium is attained in the presence of the disease, which is denoted by:

$$\mathcal{E} = (S, E, I_a, I_s, IH_m, H_c, R, D, E_{nv}) = (S^*, E^*, I_a^*, I_s^*, H_M^*, H_C^*, R^*, D^*, E_{nv}^*) \quad (18)$$

and the values of E^*, I_s^*, H_m^*, H_c^*, R^* and E_{nv}^* can be computed from the equations:

$$\pi - r_4\beta_1\left(\frac{\eta_a I_a + I_s}{N}\right)S - r_4\beta_2\left(\frac{E_{nv}}{E_{nv}+K} - \mu\right)S = 0 \quad (19)$$

$$r_4\beta_1\left(\frac{\eta_a I_a + I_s}{N}\right)S + r_4\beta_2\left(\frac{E_{nv}}{E_{nv}+K}\right)S - \sigma E - \mu E = 0 \quad (20)$$

$$(1-\theta)\sigma E - \rho I_a - \varepsilon I_a - y I_a - \mu I_a = 0 \quad (21)$$

$$\theta\sigma E + \varepsilon I_a - e I_s - \omega I_s - \alpha_1 I_s - \mu I_s = 0 \quad (22)$$

$$\rho I_a + e I_s - xIH_m - iIH_m - \mu IH_m = 0 \quad (23)$$

$$xIH_m - gH_c - \alpha_2 H_c - \mu H_c = 0 \quad (24)$$

$$gH_c + iIH_m + yI_a + \omega I_s - \mu R = 0 \quad (25)$$

$$\alpha_1 I_s + \alpha_2 H_c = 0 \quad (26)$$

$$aI_a + bI_s - \varphi E_{nv} = 0 \quad (27)$$

From Equation (21), solving for E^*, we have

$$E^* = \frac{(\rho+\varepsilon+y+\mu)}{(1-\theta)\sigma} I_a^*; \quad (28)$$

from Equation (22) solving for I_s^* we have

$$I_s^* = \frac{\theta(\rho+\varepsilon+y+\mu)+\varepsilon(1-\theta)}{(1-\theta)(e+\omega+\alpha_1+\mu)} I_a^*; \quad (29)$$

from Equation (23), solving for H_m^* we have

$$H_m^* = \frac{\rho(1-\theta)(e+\omega+\alpha_1+\mu)+e(\theta(\rho+\varepsilon+y+\mu)+(1-\theta)\varepsilon)}{(x+i+\mu)(1-\theta)(e+\omega+\alpha_1+\mu)} I_a^*; \quad (30)$$

solving for H_c^* from Equation (24) we have

$$H_c^* = \left(\frac{x}{g+\alpha_2}\right)\left(\frac{\rho(1-\theta)(e+\omega+\alpha_1+\mu)+e(\theta(\rho+\varepsilon+y+\mu)+(1-\theta)\varepsilon)}{(x+i+\mu)(1-\theta)(e+\omega+\alpha_1+\mu)}\right) I_a^*; \quad (31)$$

solving Equation (25) for R^* we have

$$R^* = \frac{g}{\mu}H_c^* + \frac{i}{\mu}IH_m^* + \frac{y}{\mu}I_a^* + \frac{\omega}{\mu}I_s^* - 0 \text{ and} \quad (32)$$

by solving Equation (27) for E_{nv}^*, we can get

$$E_{nv}^* = \frac{a(1-\theta)(e+\omega+\alpha_1+\mu)+b[\theta(\rho+\varepsilon+y+\mu)+(1-\theta)\varepsilon]}{(1-\theta)(e+\omega+\alpha_1+\mu)\varphi} I_a^*. \quad (33)$$

References

1. Wu J, Dhingra R, GambhirM, Remais JV. Sensitivity analysis of infectious disease models: methods, advances and their application. J R Soc Interface. 2013; 10.

2. Driessche PV, Watmough J. Reproduction numbers and sub-threshold endemic equilibria for compartmental models of disease transmission. Mathematical Biosciences. 2002; Volume 180, Issues 1–2.

3. Diekmann O, Heesterbeek JA. & Metz JA. On the definition and the computation of the basic reproduction ratio R_0 in models for infectious diseases in heterogeneous populations. J. Math. Biol. 1990;28,365-382 . https://doi.org/10.1007/BF00178324

4. Marino S, Hogue IB, Ray CJ, Kirschner DE. A methodology for performing global uncertainty and sensitivity analysis in systems biology. Journal of Theoretical Biology. 2008;Volume 254, Issue 1.
