# Supplementary material for: Assessing the impact of non-pharmaceutical interventions (NPI) on the dynamics of COVID-19: A mathematical modelling study of the case of Ethiopia
Source: PLoS One. 2021 Nov 16;16(11):e0259874. doi: 10.1371/journal.pone.0259874 (PMC8594814; doi:10.1371/journal.pone.0259874)
Supplement: Attachment — Submitted filename: Response to Reviewers.pdf [file pone.0259874.s003.pdf]

Editor comments.

1.) Please ensure that your manuscript meets PLOS ONE's style requirements, including those for file naming.

- Addressed.

2.) Thank you for stating the following financial disclosure:

'The funders had no role in study design, data collection, and analysis, decision to publish, or preparation of the manuscript.'

At this time, please address the following queries:

1. Please clarify the sources of funding (financial or material support) for your study. List the grants or organizations that supported your study, including funding received from your institution.
2. State what role the funders took in the study. If the funders had no role in your study, please state: "The funders had no role in study design, data collection, and analysis, decision to publish, or preparation of the manuscript."
3. If any authors received a salary from any of your funders, please state which authors and which funders.
4. If you did not receive any funding for this study, please state: "The authors received no specific funding for this work."

Please include your amended statements within your cover letter; we will change the online submission form on your behalf.

- Addressed. In the revised cover letter, the following statement included.

"This work was supported by Addis Ababa University, Ethiopia, and as a sandpit project of the One Health Regional Network for the Horn of Africa (HORN) project. The HORN project is funded by UK Research and Innovation (UKRI) and the Global Challenges Research Fund (GCRF) from the Growing Research Capability call awarded to the University of Liverpool, UK (project number BB/P027954/1). Funders had no role in the design, analysis, and interpretation of the results. Some of the Authors (Bedilu A. Ejigu, Manalebish D. Asfaw, Lisa Cavalerie, and Tilahun Abebaw) received salary."

3.) Please amend your authorship list in your manuscript file to include author Mark Nanyingi.

- Corrected.

4.) Please ensure that you refer to Figure 1 in your text as, if accepted, production will need this reference to link the reader to the figure.

- In the revised version, Figure 1 cross-referenced.

Reviewers' comments:

Reviewer #1:
This paper presents a mathematical model and analysis to investigate the impact of alternative non-pharmaceutical interventions (NPI) on the spread of COVID in Ethiopia. It builds upon a standard epidemiological model (SEIR) and adds components for symptomatic and asymptomatic infected individuals, and hospitalized individuals. It also adds a component for transmission from the environment. The model considers the impacts of NPI by essentially decreasing the transmission rates between model components. The model has 9 state variables covered by a standard system of ODEs with 22 parameters with values estimated from the limited data available or assumed. The model is used to project the dynamics of the state variables under several NPI scenarios separately for the urban and rural populations of Ethiopia.

The assessment of this paper would have been enhanced if the following issues had been addressed.

1. The introduction says "We believe that, due to the difference in the age structure of the population, social interaction and lifestyle in Ethiopia, mathematical models developed in other countries may not work to study the dynamics of disease in lower-income settings." Given the tremendous number of COVID models already developed, this statement requires justification, particularly because the model they use has nothing at all about age-structure and the manner in which the differences in lifestyle and social interactions are included in the model would be readily accounted for in standard SEIR models by simple modifications of the parameter values.

- We thankfully accept the comment about the age-structure. Initially, we assumed we would get data stratified by age. In the meantime, it was difficult to find such data, and in the revised version we amended the text accordingly. Regarding the lifestyle, urban and rural population differs in a number of ways. Such as, in rural areas, people live in a more scattered way than the urban, and the way they greet when they meet each other is also different (i.e. in rural areas there is no custom of handshaking). Further, the attention they give to hygiene is another source of differences considered in the paper.

2. The model consists of two non-linear ODEs in a standard epidemiological format, linked to a linear system of ODEs for all the other 7 state variables. The system is therefore essentially equivalent in terms of long-term dynamics to a system of three equations and standard general theory of ODEs can be applied to show the resmdeified - the paper ults of Theorem 2.1 that is

proved in the appendix, as well as the other two Theorems (indeed the authors refer to a previous paper for the proof if Theorem 2.2 and say nothing at all about Theorem 2.3 except state it. Thus the paper can be greatly reduced in length by referring to general ODE results.

- We thankfully accept the comment. Details on the mathematical model system equilibria, stability of the system, sensitivity analyses, and well-posedness of the proposed mathematical model were provided as supplementary material of the revised version.

3. There is no mention of evaluation nor is there any attempt to evaluate the model. With no criteria provided to state whether the model is appropriate, it's not clear that the projection they produce are at all meaningful. At the least, I would expect some parameterization to be chosen and compared to the available data for a portion of the time series of at a and then use to project for the later part of the time series with some criteria applied to infer the model is reasonable. As it stands the implication from Table 4 is that the core dynamics of the infection was changing rapidly over the course of the year so that it is far from clear that the model assumptions of constant parameter values is reasonable.

- We thankfully accept the comment. Because of little surveillance testing for COVID-19 in Ethiopia, it is difficult to get suitable data for model validation. Our aim in this study was to project the course of the disease in Ethiopia at a point in time depending on different adherence levels of NPIs. Due to the limited availability of data in Ethiopia we have used values from the literature to parameterize our model. However to assess the importance of considered parameters in the considered model, we have performed a sensitivity analysis by computing partial rank correlation coefficients (PRCC). Further, to see whether one parameter depends on another, pair wise comparisons were carried out.
- The difference in Ro (presented in Table 4) over the course of the dynamics is due to the intervention enforced by the government. Our model is helpful in indicating trends for projected cases depending on different levels of NPIs implementation.

4. The paper is missing key descriptions that would be required to allow repeatability of the results stated. It is really not at all clear how many parameters were estimated. For example, the virus decay rate is stated as 1/4 with no source except "average" and many of the sources of the parameters are "assumed". Similarly, the assumed differences between the urban and rural populations are assumed to be very specific values (27.4% and 7.8% of the urban and rural populations wash their hands) with little assessment that these could be tremendously off. There is no code listed or methods described for the dynamic solutions of the systems of equations in the model -m presumably they are using some standard ODE solver but they do not say.

- We thankfully accept the comment and in the revised version, sources were explicitly mentioned (Table 1, last column), and additional references were included in the reference list.
- A number of factors (temperatures, relative humidity, and UV Index) influence the COVID-19 virus decay rate and we consider the average. In the revised manuscript references were given.
- The assumed difference between the urban and rural populations (27.4% and 7.8% of the urban and rural populations wash their hands) is evidence based as revealed by the 2016 Ethiopian demographic and health survey. Reference sources included in the revised version of the manuscript.
- For repeatability of the results, **matlab** codes used to generate the results in the manuscript and now included in the supplementary material.

5. There is no discussion of data quality in the data sets on the disease progression, and in fact it isn't clear how these data sets were utilized in the model analysis. Given the variety of concerns about reporting inadequacies in many countries, some discussion of the implications of poor data should be included.

- We have used publicly available data (i.e. daily number of new cases, deaths) and some variables (i.e. daily number of ICU cases) extracted from ministry of health and Ethiopian public health institute reports. The testing rate is indeed quite low in Ethiopia compared to other countries, however given the fact that the number of cases is only uses as indicative trend prior to our projection, this has little consequences on our model projections
- We mentioned the impact of poor data on the "Limitation" subsection of the manuscript. The testing rate of COVID-19 is very low in Ethiopia which may lead the number reported of cases is underestimated.

6. The main results in Fg 10 on the sensitivity analysis are completely obvious from the formula for R0 given that R0 has a factor of r4 which has symmetric negative dependence on SD, c FM and HW. Similarly, the dependence on Beta1 enters because Beta1is a factor in the two terms of the R0.

- Yes that is true. But, for the general audience, the equation for Ro is not obvious. Further, the sensitivity analysis graph is a better visualization guide for future efforts to better describe the impact of hygiene, social distancing and wearing facemask. In addition to this, this section also tries to investigate whether one parameter depends on another using a pair wise comparison test.

7. Major portion of the manuscript is in Figures 2-9 which illustrate the dynamics of the model for the rural or urban population under a limited set of scenarios for NPI but there is no

justification given for the scenarios chosen so the reader has no way of asserting that these are scenarios which are indeed more meaningful than the many other ones that are possible.

- We thankfully accept the comment, and in the revised manuscript, the reason for considering those scenarios was given in the revised method Section 2.3 as follow. “These values were selected based on expert opinion of how the dynamics may look under the implementation of NPIs with different adherence levels in the urban and rural populations. Further, the considered NPI’s coverage is supported by phone-based survey results. (Baye 2020, Kebede et al 2020).”

8. There are many, many places where the grammar and sentence structure needs to be modified. Similarly, many of the references are not inappropriate style. Figure 1 is confusing - some dashed lines have arrows and others do not and it isn't clear why some are dashed and some are not based on the equations - the Env equation has a dashed line from S for some reason. The notation for the state variable IHm is confusing since it seems to be two variable

- Flow diagram of the mathematical model showing the transition of individuals in different compartments based on infectious status (solid lines). The model also includes the environment, which receives infection from asymptomatic and symptomatic individuals and from which infection can pass to susceptible individuals (dashed lines), at a rate dependent on the force of infection.
- To avoid confusing, the state variable IH_m changed to H_m.

9. The authors assume that there is no movement of individuals at all between the urban and rural populations. This justifies their efforts to look at the NPI in the two separately. However, this assumption needs to be justified.

- Your concern in this respect is valid. We draw this assumption due to the fact that there was very low road accessibility to connect the urban and rural population of Ethiopia (World-Bank (2016). Measuring Rural Access).
