# Supplementary material for: Assessing the impact of non-pharmaceutical interventions (NPI) on the dynamics of COVID-19: A mathematical modelling study of the case of Ethiopia
Source: PLoS One. 2021 Nov 16;16(11):e0259874. doi: 10.1371/journal.pone.0259874 (PMC8594814; doi:10.1371/journal.pone.0259874)
Supplement: Attachment — Submitted filename: Response to Reviewers.pdf [file pone.0259874.s004.pdf]

Responses to reviewer's comments

Review Comments to the Author

Reviewer #1: The authors have added a supplementary information component to this paper that improves the clarity of the mathematical results.

The manuscript does not state what numerical method they are using in the Matlab code - it appears they are using the Euler method and some justification why a more standard ODE solver within Matlab is not provided. Some justification for the numerical stability of the Euler method they implement is needed since there are many reasons why this method is replaced by others. Some of the rate parameters in the model are one or two orders of magnitude different from other rate parameters, which implies the potential for stiffness, which is one of the conditions under which the Euler method is unstable numerically.

- There are several methods for solving ODEs. We prefer to use Euler because Euler method is used in solving differential equations with known initial conditions. Further this method also used to approximate the behavior of the equations involved in a certain range. To increase the accuracy of the method we used a time step of 0.01.

In response to comments regarding model evaluation (review comment #3), the authors still make no attempt to compare the model results with data. They state that the sensitivity analysis provides insight into the importance of model parameters, which it does, but sensitivity analysis provides no evidence in and of itself that the model assumptions and results appropriately describe the dynamics of the system so as to be useful.

- In the revised version of the manuscript, we did the model evaluation using the actual data in the month of October. The following figure shows how the predicted number of cases (red line) looks as compared with the actual reported case by ministry of health (blue line). The result reveals that the predicted number of cases by the proposed model are a bit higher than the actual cases. This may be due to under reported cases because of very low test coverage in Ethiopia. Further, the difference between the actual reported number of cases and predicted cases ranges from 2053 to 6946 individuals which is acceptable prediction in error in a country with a large population (above 110 million in the case of Ethiopia). The two lines have similar slopes, and that the numbers of cases are similar given the margins of error and likely under-detection.
- Under moderate implementation of NPIs (20% -social distancing, 5%-face mask coverage, and 25% hand wash), we compared predicted cases of with data available from the ministry of health. The predicted and observed cases are shown in Fig 11. The predicted cases are very close to those observed, and there is a similar slope, indicating that our model is successfully capturing the dynamics of Covid-19 in Ethiopia.

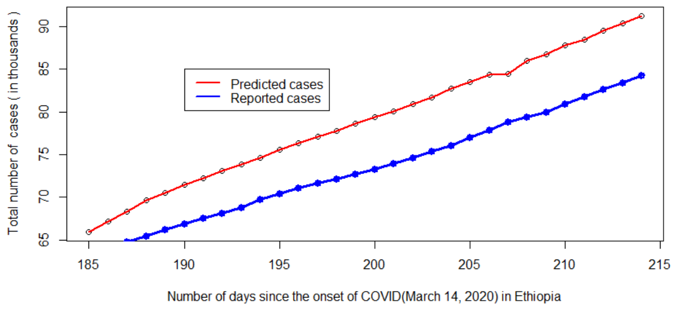

In response to review comment #3 the authors state that the results in Table 4 arose from different "interventions enforced by the government" over the time period modeled. However there is no statement about government timing of interventions in the data section. It appears Table 4 gives model results assumed to be from the interventions and adherence levels given in Table 2, but there is no mention of the timing of these interventions. So if the interventions start at the beginning of the time period and remain constant, why are the R_0s changing so much over time in the model results in Table 4?

- Yes, the interventions start at the beginning of the time period and remain constant, while the adherence level in the population varies over time. For instance, in the week after the holidays of Easter and Ramadan (61-90 days since the onset of COVID in Ethiopia), there was increased travelling and intense interaction in the population. Moreover only schools remain closed, and social distancing in public transport and market places wasn't adhered to. The government was able to support and enforce wearing of face masks in public but, due to socioeconomic constraints, was less able to enforce adherence to social distancing and hand washing.
- The MATLAB code calculates the new Ro considering time-varying levels of adherence to the NPIs. The MATLAB code included in the supplement.

The references in the revision are still not formatted consistently.

- Addressed.

This article has still not been copyedited and since PLOS does not copy edit manuscripts this manuscript still needs to be corrected in many places for grammatical reasons.

- Addressed. The revised manuscript edited by native speaker.

Figure 1 is still unclear - there are still dashed lines with no arrows and one dashed line with an arrow - what do the dashed lines represent? The arrows are still left out.

- In Figure 1 we have two main compartments: i) Human population and ii) Environment. To delineate the human to human (H2H) interaction and progression from different states (SEIR) we used solid lines. The interaction of these human compartments with the environment are indicated by broken lines. The dashed lines represent potential shedding of the virus to the environment by individuals in the

asymptomatic and symptomatic compartments, the susceptible population may be infected from the contaminated environment. Such schematic representations has been used in modeling infectious diseases involving contaminated environment such as malaria and cholera.

Table 1 - the values are a mixture of fraction and decimal notation - these should be uniform.

- Addressed

Supplementary information Comments

1. In Theorem 0.1 there is a mention of Theorem 2.1 after the Theorem statement - what is this in reference to?
   - Thanks for pointing out this. It was a typo error and corrected in the revised version.

2. Sensitivity analysis section states: The partial derivative of the threshold value Ro with respect to the input parameters were computed by varying the parameters around normal values.What are "normal values".

   - These values were obtained from the table of parameters the sensitivity analysis studies what would happen if we perturb the values within a certain interval.

3. In the paragraph after eq (11) there is a [?] ref
   - A reference has been inserted.

4. The Matlab codes provided have no overall description, nor do they appear to include the all calculations discussed in the paper - for example there does not appear to be any sensitivity analysis included in the Matlab codes

   - In the revised manuscript, a brief description of the Matlab codes is included. The sensitivity analysis code has also been included in the attached Matlab code.

5. The references in the supplement are not in standard uniform style.
   All references have been updated using a uniform bibliographic format.
