# Supplementary material for: Assessing the impact of non-pharmaceutical interventions (NPI) on the dynamics of COVID-19: A mathematical modelling study of the case of Ethiopia
Source: PLoS One. 2021 Nov 16;16(11):e0259874. doi: 10.1371/journal.pone.0259874 (PMC8594814; doi:10.1371/journal.pone.0259874)
Supplement: Attachment — Submitted filename: PONE_D_20_37247-R3.pdf [file pone.0259874.s005.pdf]

Review of the manuscript entitled ***Assessing the impact of non-pharmaceutical interventions (NPI) on the dynamics of COVID-19: a mathematical modelling study of the case of Ethiopia***.
Authors: Bedilu Alamirie Ejigu, Manalebish Debalike Asfaw, Lisa Cavalerie, Tilahun Abebaw, Mark Nanyingi, Matthew Baylis
Journal: PLOS ONE
Manuscript ID: PONE-D-20-37247-R3

The article concerns a mathematical modelling approach to investigate the dynamics of COVID-19 as a non-pharmaceutical interventions. It focuses on the COVID-19 pandemic in Ethiopia by implementing the model based on the real data. Moreover, the model predicts the severity of COVID-19 with respect to the non-pharmaceutical interventions from the viewpoint of Ethiopian public health system. The results presented in the manuscript are interesting and exciting as well. In my opinion, the manuscript can be accepted for publication after few minor issues mentioned below.

Major Corrections:

1. Recommendation: **Accept**
2. Is the manuscript technically sound, and do the data support the conclusions? - **Yes**
3. Has the statistical analysis been performed appropriately and rigorously? - **I don't know**
4. Does the manuscript adhere to the PLOS Data Policy? -**N/A**
5. Is the manuscript presented in an intelligible fashion and written in standard English? - **Yes**
6. Review Comments to the Author:
   (a) The quality of all the presented figures should be improved.
   (b) More recent references regarding the Mathematical Modelling of COVID-19 should be included.
   (c) A brief description of determining the basic reproduction number R_0 is needed.
7. Would you like your identity revealed to the authors of this submission? - **No**
8. Do you have any potentially competing interests? - **None**
